# Relating Neuronal to Behavioral Performance: Variability of Optomotor Responses in the Blowfly

**DOI:** 10.1371/journal.pone.0026886

**Published:** 2011-10-31

**Authors:** Ronny Rosner, Anne-Kathrin Warzecha

**Affiliations:** 1 Department of Biology, Philipps-University Marburg, Marburg, Germany; 2 Department of Neurobiology, Bielefeld University, Bielefeld, Germany; Lund University, Sweden

## Abstract

Behavioral responses of an animal vary even when they are elicited by the same stimulus. This variability is due to stochastic processes within the nervous system and to the changing internal states of the animal. To what extent does the variability of neuronal responses account for the overall variability at the behavioral level? To address this question we evaluate the neuronal variability at the output stage of the blowfly's (*Calliphora vicina*) visual system by recording from motion-sensitive interneurons mediating head optomotor responses. By means of a simple modelling approach representing the sensory-motor transformation, we predict head movements on the basis of the recorded responses of motion-sensitive neurons and compare the variability of the predicted head movements with that of the observed ones. Large gain changes of optomotor head movements have previously been shown to go along with changes in the animals' activity state. Our modelling approach substantiates that these gain changes are imposed downstream of the motion-sensitive neurons of the visual system. Moreover, since predicted head movements are clearly more reliable than those actually observed, we conclude that substantial variability is introduced downstream of the visual system.

## Introduction

Behavioral responses of animals to repeated presentation of the same stimulus usually vary from trial to trial. This variability can be due to changes of the internal state of the animal as well as to stochastic processes underlying information processing in the nervous system and in the motor system eventually producing behavior. Decisive sources of variability are tackled in a range of animal systems [1]. For instance, most of the noise affecting smooth pursuit eye movements in monkeys has been concluded to arise already in the sensory processing stages [2]. There is ongoing controversy on this topic as other studies of primate eye movements demonstrated that motor noise exceeds the sensory noise [3], [4]. In the visual system of insects the issue of neuronal ([5]–[10], reviews: [11]–[14]) and behavioral [15], [16] variability has been addressed. However, even in these systems the decisive sources for behavioral variability have not yet been located.

In the present paper we analyse whether neuronal variability at the output level of the visual motion pathway can cause the observed variability in the final behavior. We use visually induced head pitch movements of blowflies as our experimental paradigm. The considerable variability of these movements is largely due to two different motor activity states in the fly [16]. However, even within one activity state the responses are variable. The activity state also affects the responses of motion-sensitive neurons in the fly's visual system, the so-called lobula-plate tangential cells (LPTCs) [17]–[20], as well as a subsequent motor neuron [21]. LPTCs, which can be identified individually based on their physiological and anatomical properties, encode motion information within large parts of the visual field [11], [22]–[24]. The LPTCs mediating downward directed head pitch movements are directly presynaptic to the motor neurons innervating the respective neck muscles [25], [26]. It has been suggested that the state associated optomotor gain changes are brought about by a central signal downstream of the visual system [16], [17], [21]. Because of its straightforwardness the system investigated here is particularly well-suited to analysing whether the variability found at the sensory level can account for the variability of the behavioral output.

By comparing neuronal and behavioral responses via a simple modelling approach we conclude that the variability present at the level of LPTCs is too small to account for the variability of head pitch responses both within and across behavioral states.

## Results

We aim to discover whether the variability found at the level of motion-sensitive LPTCs can account for the variability of head optomotor pitch responses or, alternatively, whether variability comes into play downstream of the LPTCs. We used neuronal responses of VS2/3-cells that mediate head pitch movements [25], [26] to predict head movements by applying a simple modelling approach. We then compared the reliability of predicted and actual head movements.

**Figure 1:**
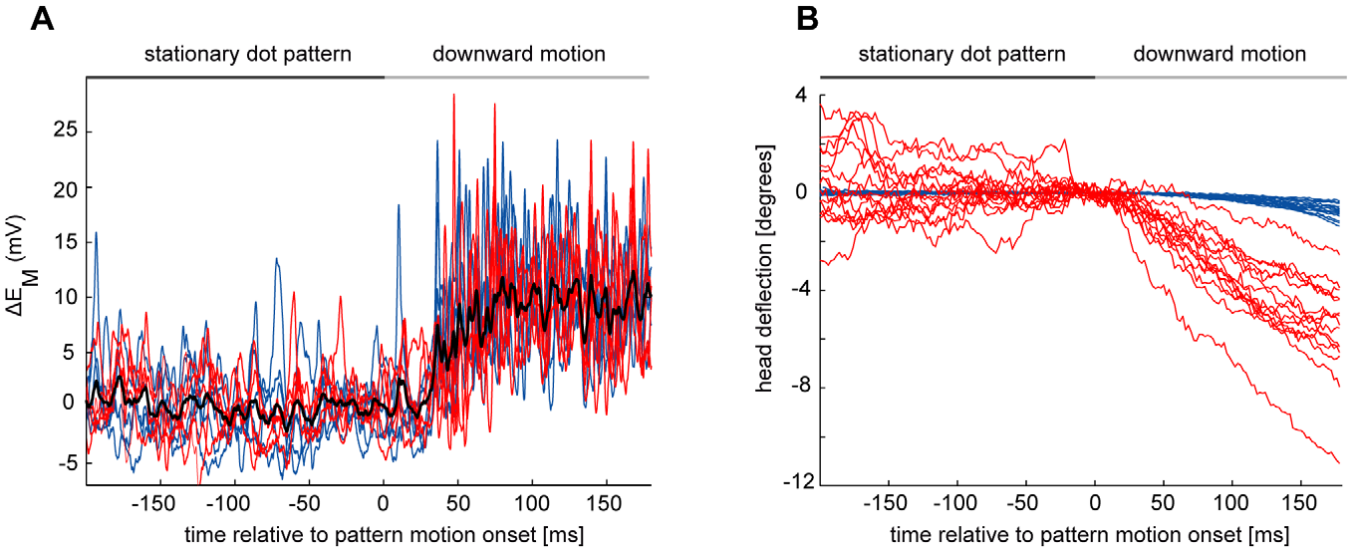
Variability of neuronal and behavioral responses to downward motion during the two motor activity states. (A) Ten individual trials of membrane potential changes of a VS2/3-cell in the reduced (blue) and elevated (red) motor activity state and mean across trials (black) illustrating step-like response profile. Membrane potential is shown relative to the resting potential measured while no pattern was presented. (B) Fifteen individual head pitch responses during reduced (blue) and elevated (red) motor activity of another fly (for details of the analysis see [16]). The VS2/3-cell responds with a sudden change of the membrane potential and stays depolarised throughout motion stimulation whereas the head pitches slowly downwards yielding an inverted ramp-shaped response profile. The behavioral responses show a conspicuous state dependence while the neuronal responses do not. Note that the variability of the traces is largely reduced around time 0, because of the analysing procedure (see Materials and Methods section).

VS2/3-cells respond to the onset of constant velocity motion with a sudden depolarisation of their membrane potential (example traces in Fig. 1A). The membrane potential stays depolarised throughout the presentation of the motion stimulus. In these cells the graded membrane potential change is often superimposed by spikes of variable amplitude [27], [28]. VS-cell responses to repeated presentations of an identical motion stimulus differ from one trial to the next (Fig. 1A). Large parts of this variability could be caused by different behavioral states of the animal (review: [29]). As an indicator of the activity state we examined the movement of the halteres which are known to oscillate when a fly walks or flies [30]. However, the neuronal responses elicited by strong visual stimulation did not clearly depend on the state of motor activity of *Calliphora* (Fig.1A). Possible reasons for the insignificant state-dependence are discussed below. Even during the presentation of the stationary dot pattern prior to pattern motion onset, we did not observe clear state-dependent changes of VS2/3 cell responses.

Like the neuronal responses, head pitch responses elicited by the same motion stimulus also differ from trial to trial (Fig. 1B). Compared to the basically step-like neuronal responses, head pitch responses are ramp-shaped, changing throughout the entire recorded time period in response to constant velocity motion (Fig. 1B). VS2/3 cells depolarise during downward motion and, as a consequence, the head bends downward reducing the slip velocity on the retina. In contrast to the neuronal responses, the head pitch responses take a conspicuously different gain depending on the state of motor activity. If the fly is in an elevated motor activity state, the optomotor gain is much higher than in the inactive state of the fly (Fig.1B; [16]).

To directly relate the variability at the neuronal to that at the behavioral level we modelled the sensory-motor transformation by applying a low-pass filter to the neuronal responses in order to then compare predicted and measured head pitch (for rationale of low-pass filter approach see Discussion section).

**Figure 2:**
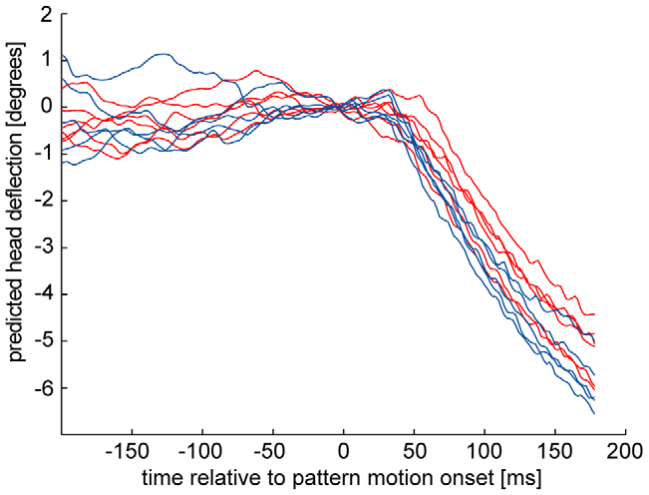
Head pitch movements predicted from neuronal responses to downward motion. A first-order low-pass filter (τ = 150 ms) was applied to neuronal responses of a VS2/3-cell recorded in close temporal succession during the presence (red) or absence (blue) of haltere oscillations.

The prediction of head pitch movements from neuronal responses recorded in either the elevated or the reduced motor activity state yields very similar results (Fig.2) and does not match the findings at the behavioral level with respect to the large state-dependent gain changes observed there. Since in accordance with previous suggestions [16], [17], [21] the drastic state-dependent head optomotor gain changes cannot be explained by the LPTC responses, we implement a gain modulating mechanism downstream of the LPTCs. This adjusts the optomotor gain to the particular state of motor activity. Because of the apparent lack of state-dependence in our neuronal data all stable electrophysiological recordings (see Materials and Methods section for definition of stability) were used for predicting head movements independent of whether they were recorded during or without haltere activity. Note that, if there actually were any differences between the neuronal responses obtained in the high versus low activity state our procedure should increase the variability of the predicted head pitch responses. As we will see later, overestimating neuronal response variability does not affect our conclusions.

**Figure 3:**
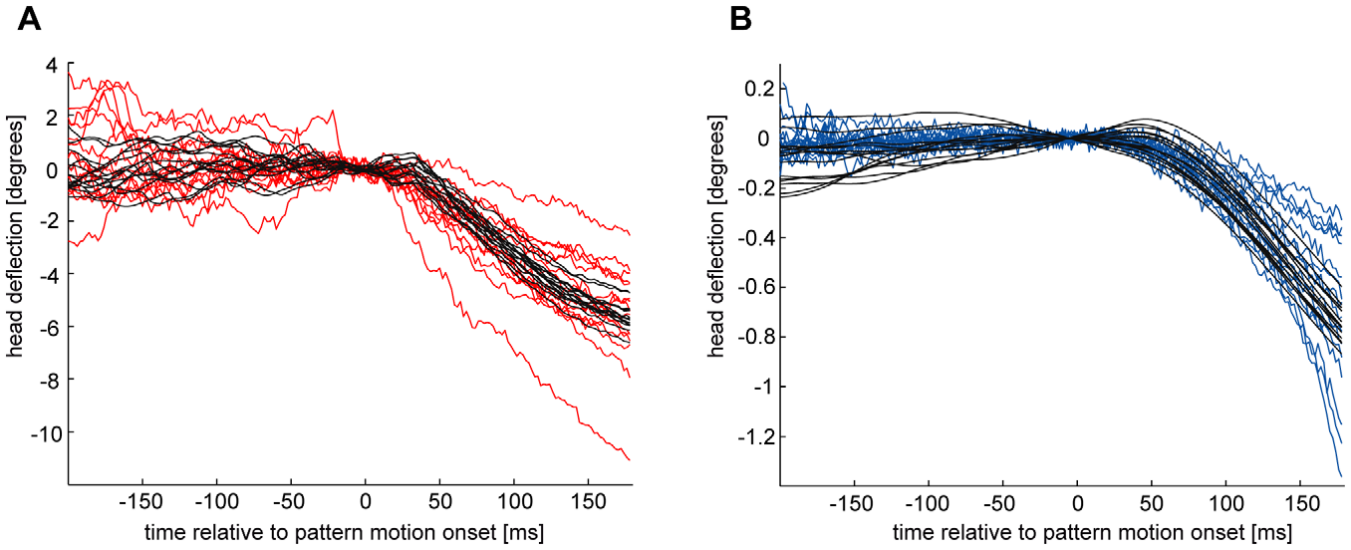
Comparison of predicted and actually observed head pitch responses to downward motion. The same neuronal responses were used for the predictions in (A) and (B). The gain factor was adjusted to fit the mean response amplitude of the behavioral responses. (A) Behavioral responses (red) were recorded while the fly was in a state of elevated motor activity. A first-order low-pass filter (τ = 100 ms) was used to predict head pitch from neuronal responses. Predictions (dark gray) are much less variable than the actually observed responses. All pitch responses recorded in the elevated activity state of one fly are shown. (B) Neuronal and behavioral (blue) responses were recorded while the fly was in a state of reduced motor activity. A second-order low-pass filter (τ_1_ = 100 ms, τ_2_ = 100 ms) better approximates head pitch in the reduced motor activity state than a first-order filter. Again, predictions (dark gray) are less variable than the actually observed responses. Only a subset of the recorded traces is shown for illustration of response shape and variability. Note the different scales in (A) and (B).

For predicting head movements from VS2/3-cell responses we chose three different time constants (τ_1_ = 50 ms; τ_2_ = 100 ms; τ_3_ = 150 ms) for the low-pass filter, in order to cover a range of temporal properties of the filtering algorithm and also to test to what extent the results depend on them. Between the two states of motor activity, not only the gain but also the shape of the time-dependent responses differs (Fig. 3 and [16]). Head pitch movements in the high activity state are well approximated by first-order low-pass filtering the neuronal responses (Fig. 3A). However, head pitch traces recorded while flies were in the low motor activity state become steeper throughout the evaluation period (Fig. 1B and [16]). This response profile can be approximated better by filtering neuronal responses with a second-order low-pass filter (Fig. 3B). It is not unreasonable to assume such a filter because it has been suggested that resting flies actively pull their head to the thorax [31]. Such a mechanism implies a second damping process in the reduced activity state of the fly acting upon the already existing low-pass properties of the neck motor system. Fig. 3B shows several example predictions of head pitch movements derived from filtering neuronal responses of a single cell. The overall shape of the predicted responses meets the observed ones very well. Hence, in the following a first-order low-pass filter and a high gain were used to predict head pitch responses in the high activity state; to predict head pitch movements observed in the low activity state a second low-pass filter was applied in conjunction with a low gain.

**Figure 4:**
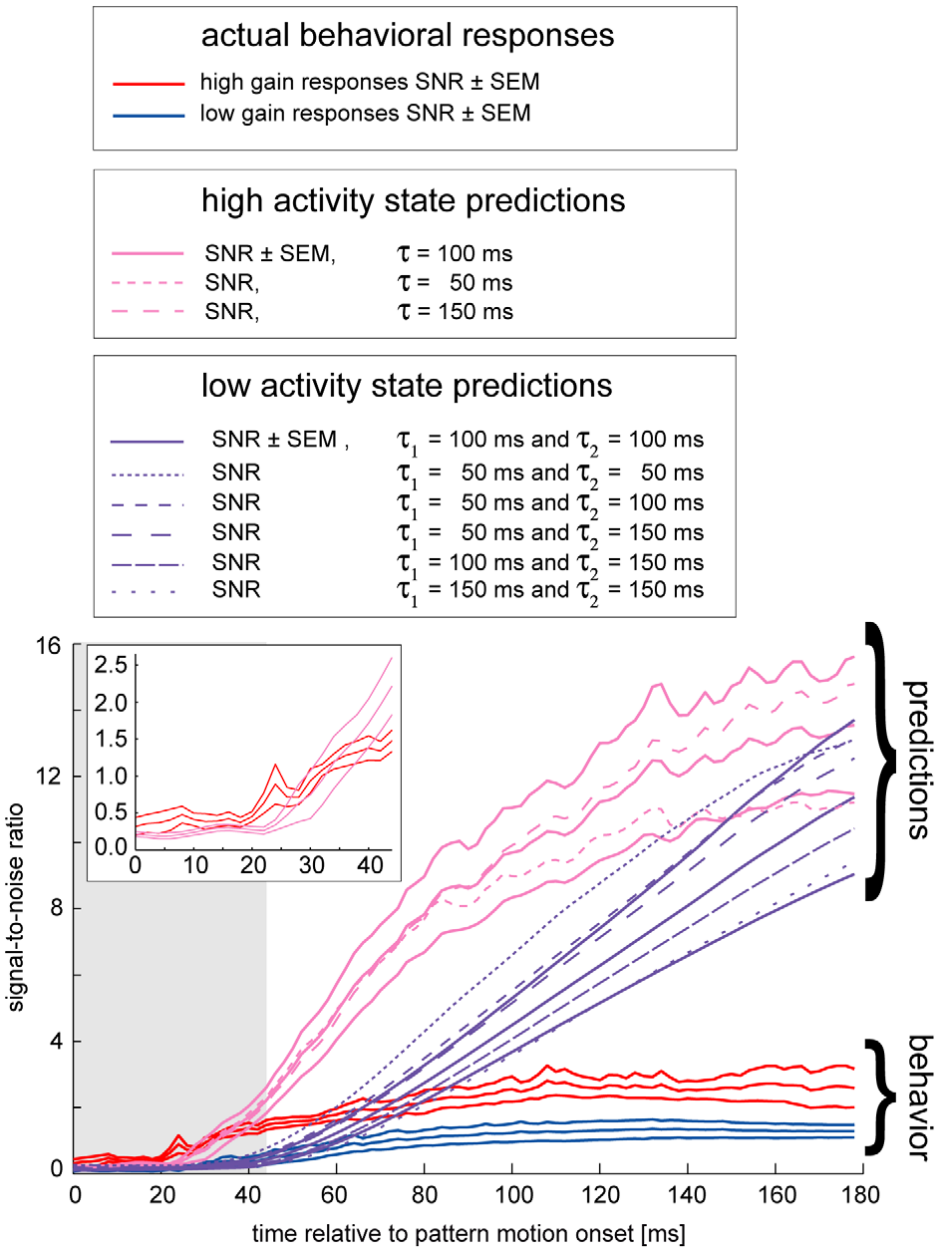
Signal-to-noise ratios for simulated and actually observed head optomotor pitch movements. Signal-to-noise ratios (SNRs) are means across flies (predictions: N = 7, behavior: N = 6) and plotted as functions of time after stimulus motion onset. In order to predict head pitch in the elevated (reduced) motor activity state a first-order (second-order) low-pass filter was used. The SNR of actually observed head movements at the end of the trials in both motor activity states is considerably smaller than predicted for the respective state. At the end of the open-loop interval (grey shaded box and inset), the SNR of the predicted high activity state responses already outreaches the SNR of the head movements recorded in that state (see text for details). Note that the seeming state difference in SNRs of actually observed responses is at least in part the consequence of spontaneous head pitch superimposing on the visually induced response (see [16]).

As a measure of the reliability of the predicted and the experimentally determined pitch responses we calculated the signal-to-noise ratio (SNR) as a function of time (Fig. 4). The SNR is determined as the ratio of the mean head deflection at a given time (i.e. across trials) divided by the corresponding standard deviation. The SNR of the observed head pitch responses first increases and then tends to saturate about 100 ms after motion onset for both the high and the low activity state. In comparison, the SNRs of the predicted responses also indicate some saturating behavior but do not reach their plateau values within the analysed time interval. Importantly, the SNRs of the predicted responses for the high and low motor activity state are higher within large parts of the evaluation period than the SNR of the actually measured head movements. The difference in the SNRs of the predictions and the observed optomotor responses increases with time. Which time period should be consulted for comparing predicted (neuronal) and measured (behavioral) SNRs? On the one hand a late point in time is preferable for obtaining a reliable comparison of the SNRs because the early phase of the behavioral responses is largely the consequence of the condition of the neuronal machinery and the neck-motor system preceding the downward-directed motion stimulus. Head movements preceding the motion stimulus, however, can be expected not to be dominated by VS2/3-cell responses, because at that time their response strength should not be exceptionally large compared to other input channels influencing the neck-motor machinery (see Discussion). In the present study, however, we want to analyse optomotor responses that are mediated by VS2/3-cell responses and accordingly our model only takes VS2/3-cell responses into account. Predictions and behavioral responses should therefore be compared when the influence of the VS2/3-cell response on the neck motor machinery is clearly noticeable.

At the last evaluated data points, 178 ms after stimulus motion onset, the SNR of the VS-cell-based predictions (S/N_sens_) clearly surpasses the SNR of the observed behavioral responses (S/N_behav_) for both the high and low activity state (Fig.4). This result suggests that noise is added after sensory signal processing, i.e. beyond the LPTCs. The following thoughts allow us to estimate the amplitude of this postsensory noise relative to the noise observed in the LPTCs:

When assuming independent normally distributed noise for both the responses of the VS2/3 cells (N_sens_) as well as for the downstream, postsensory processing (N_post_), the variances of these two parameters add to yield the variance of the final behavioral head pitch (N^2^_behav_). Hence the variance of the postsensory noise can be estimated according to the following equations: 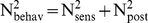
(1)


The predicted SNR (S/Nsens) can be related to the SNR of the observed head pitch movements (S/N_behav_) according to: 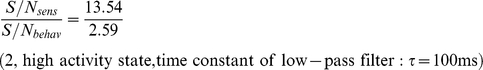



The signal S is modelled to be the same and hence cancels out:
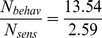
(3)


Substituting N_behav_ from (3) in (1) yields:
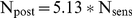
(4)


From this reasoning it follows that the variability added after the visual system is considerably larger than the sensory variability inherited from the noisy signals of the VS-cells. The chosen time constant does not affect this result (the multiplier in equation (4) for a time constant of 50 ms and 150 ms changes to 4.21 and 5.62, respectively). Taking the low activity state responses as a basis for estimating the postsensory noise one gains even higher factors.

Although a late point of time is preferablefor the aforementioned reasons, there is one important drawback, for the high activity state: Stimulus motion was identical in the experiments recording neuronal and behavioral responses. Nevertheless, the actual retinal pattern velocity was not the same in both situations. In the high activity state the head movements compensate for about 20% of the pattern velocity [16], reducing the net pattern velocity on the eyes of the fly. In other words, the loop between the sensory input and the motor output was closed in the behavioral experiments. This problem only applies to the high activity state because in the low motor activity state head movements are so small that they do not effectively impact the retinal pattern velocity imposed by stimulus motion. For the high activity state, neuronal and behavioral performance can be easily compared only within the early response (“open-loop”) phase in which the optomotor response is influenced only by the motion stimulus and not yet influenced by changes of the retinal stimulus velocity due to the fly's pitch response. However, towards the end of the open-loop phase, which is commonly assumed to correspond to twice the latency [32], the SNR of the predicted responses is already higher than the SNR of the actually observed head pitch responses. The latency of the behavioral responses was at least 22 ms [16]. Therefore the open-loop period ends approximately 44 ms after stimulus motion onset. Here the SNR of the predicted pitch responses already surpasses the SNR of the measured pitch responses. Calculating the postsynaptic noise for that moment of the high activity state (according to equations 1–4) yields a multiplier of 1.12 (1.20/1.03) with respect to the noise inherited from the VS2/3 cells for a filter time constant of 100 ms (50 ms/150 ms). This result again indicates that variability of a relevant degree is added on the way from the LPTCs to the final behavioral output. Our results are robust against the selection of filters with different time constants. Hence, we conclude that variability comes into play downstream of the LPTCs for both the reduced and the elevated motor activity state based on a linear model to predict head pitch responses.

## Discussion

In the present study we evaluated whether variability occurring in neurons at the output of the visual motion system in flies can account for the variability of head optomotor responses. We modelled the sensory-motor transformation and in this way predicted head pitch movements from responses of motion-sensitive neurons (LPTCs) that mediate this behavior. We found that (i) the tremendous difference in head optomotor gain associated with two different behavioral states cannot be explained by gain changes at the level of LPTCs. (ii) The variability of measured head optomotor pitch movements within a given activity state is higher than is expected from the variability of the VS2- and VS3-cell, the LPTCs we recorded from that mediate head pitch. Hence, downstream of the LPTCs the gain changes depending on the fly's activity state and additional variability comes into play. In the following these two findings will be related to the results of other studies and discussed with respect to their functional implications.

### Addressing the optomotor gain shift

We assume a mechanism exists which changes the gain of the investigated head movements downstream of the VS2- and VS3-cell, dependent on the fly's activity state, in accordance with what has been proposed previously [16], [17], [21] -despite the observation of state-dependent neuronal response changes at the level of LPTCs in calliphorid flies [7], [17], [20], [33] and *Drosophila* [18], [19]. Whereas head pitch movements depend considerably on the flies' activity state (Fig.1b and [16]) we did not observe obvious state-dependent response changes in VS2/3-cells during identical stimulation (Fig.2). This could be misconceived as contradicting the state-dependent changes in fly LPTCs mentioned above. However, we already concluded previously [17], that during strong visual stimulation the activity state does not substantially affect the responses of LPTCs. This was confirmed in a recent study in which responses of a spiking LPTC were recorded during tethered flight [20]. This latter study also provided evidence, that strong state dependencies are barely visible in the early phase of the response to a sudden motion onset (i.e. the transient response phase (e.g. Fig.4 in [20]). In the present study we only evaluated responses during such transient response phases. Furthermore even during the presentation of the stationary pattern a shift in the membrane potential does not always occur in LPTCs (see Fig.1B in [17]). Hence, the absence of strong state-dependent changes in the LPTCs under the stimulus conditions of our experiments is not in conflict with what was already found in previous studies. Moreover, differences in the amount of state dependent response changes could be due to the larger behavioral repertoire still available to the flies in other studies [18]–[20] compared to our experiments. In our experiments the flies could only swing halteres while in the other studies the animals could fly or walk. Most important for the current conclusions however is the following consideration: The primary goal here is not to analyse the exact quantity of changes in the neuronal response of LPTCs during different activity states of the animals but to determine whether the neuronal variability at the level of LPTCs suffices to explain the behavioral variability. As mentioned above we acquired our neuronal and behavioral data on the same kind of preparation using animals that could neither fly nor walk. Hence, if the largely restrained fly in our preparation displays a different kind of state dependence than the dependence described in flying or walking flies, this difference does not weaken our conclusion that neuronal variability of the VS2/3 cells is too small to account for the variability of head pitch movements.

If LPTC responses do not exhibit a clear activity-dependent gain change under our experimental conditions but head movements do, what then is the site of action of the gain-changing signal? The VS2- and VS3-cells are directly coupled to the motor neurons innervating the neck muscles that mediate head pitch movements [25], [26]. We hence suggest that the gain-changing signal acts at the level of the motor neurons or muscles (see below and [16], [17]). There is evidence for gain modulation at the level of the motor neurons mediating fly head movements [21], [34]. It has been proposed that a central signal changes the gain of optomotor head pitch [16], [17]. This central signal could elevate the gain of the optomotor response and, in parallel, induce the fly to walk or fly. Further evidence for such a central signal has recently been provided for head yaw [21]. However, the gain-changing signal for head pitch does not necessarily need to act on motor neurons–at least not exclusively. The signal could (additionally) release a clamp pulling the head to the thorax. Such a clamp was found to exist in the flesh-fly, *Neobellieria bullata* [31]. This clamping mechanism in the resting flesh-fly prevents proprioceptive signals about head deflections from eliciting head movements and is thought to be achieved by identified muscles that pull the head to the trunk [26], [31]. In *Calliphora* such a potential clamp could be released by the aforementioned central signal when the fly switches to a state of enhanced motor activity. This hypothesis is compatible with our finding that the time course of visually induced head movements in the low activity state is better predicted from the neuronal responses by applying a second- rather than a first-order low-pass filter. The state-dependent differences in the time course of the head movements thus suggest an additional damping process in accordance with a clamping mechanism.

### Addressing the reliability of optomotor responses

In the following we often will refer to the variability intrinsic to a given activity state as noise. Note however, that even within a given activity state, the variability of behavioral responses may in part be due to active processes of the nervous system rather than to random fluctuations [35]. We conclude that the variability of optomotor head pitch responses within a given activity state is substantially governed by variability added downstream to LPTCs based on the comparison of the optomotor response SNRs of two activity states with predictions of optomotor responses from neuronal data. To predict state-dependent movements we did not separate the neuronal data into high and low activity state responses because state dependent changes in VS2/3-cells during the visual stimulation employed here are at most very small (Fig. 2). If we thereby missed genuine state-dependent changes of LPTC responses we would overestimate sensory variability present in these neurons. Overestimating sensory response variability, however, strengthens rather than vitiates our conclusion of additional downstream variability, because such an overestimate reduces the predicted SNR which we nevertheless found to surpass the behavioral SNR (Fig4).

Although there is one VS2- and one VS3-cell in each brain hemisphere, we based our predictions solely on the responses of one neuron at a time, for reasons of simplicity. The responses of the VS2- and VS3-cell to identical large-field stimulation are very similar due to their common input organization and electrical coupling [36], [37]. Since the VS-cells from the two eyes are indirectly coupled via the V1- and VS1-cells [38], a weak correlation between the corresponding VS2/3-cells of the right and left hemisphere is plausible. Nevertheless, because of at least partly independent noise in these VS-cells, pooling their signals should further increase the SNR of the predicted head movements, even augmenting its superiority over the actually measured behavioral responses.

The average head pitch responses can well be described by linearly filtering VS-cell responses (Fig.3). Nevertheless, a reduction of the SNR downstream of the LPTCs could in principle be due to postsensory processing that differentially affects the signal and the noise as can be the case for nonlinear mechanisms. However, results from other studies indicate that responses of the frontal VS-cell responses are generally speaking transformed linearly to their postsynaptic follower neurons [39]–[41]; see also [42]. Findings of nonlinear processing in descending neurons downstream of LPTCs refer to nonlinear summation of input signals from multiple cells covering different parts of the visual field that converge onto a downstream neuron [43]. This is different from what we aim to cover with our low-pass filter approach simulating the information flow within a single sensory-motor strand. Moreover, optomotor responses have also previously been modelled by low-pass filtering of the responses of motion-sensitive neurons [15], [44], [45]. Furthermore, our approach is supported by the properties of insect muscle contractions that have successfully been modelled by applying simple low-pass filters [46]. Head deflections basically reflect the outcome of muscle contractions which are caused by neck motor neurons. Hence, applying of a low-pass filter for predicting head movements from LPTC responses is a reasonable procedure. Linear processing, however, does not affect the SNR. Therefore, we conclude that the reduction of the SNR is rather caused by additional variability impinging on the signal downstream of LPTCs.

Neck motor neurons and descending neurons integrate input from several sensory channels and from a central input reflecting the behavioral state of the fly [21], [34], [42], [47], [48]. In the present study we only provided visual stimulation leaving other sensory channels without input. However, these channels were still prone to variability, which can be expected to influence the motor neurons responsible for head pitch movements and thus to contribute to the behavioral variability. Similarly, changes in the internal state of the animal that were not taken into account by separating head movements in those occurring in the high and low activity state, respectively, may increase behavioral variability. Moreover, not only the input to the motor neurons, but also the motor neurons themselves can add variability [49], [50]. Even further downstream, muscles can introduce a considerable amount of variability as is for instance found in the medicinal leech [51]. Finally, muscles not directly involved in controlling head pitch movements as well as the respective upstream elements could add variability. For instance, lateral VS-cells, as well as the subsequent descending and motor neurons evoke head roll possibly combined with a pitch component [25], [26], [52]. By recording the responses of motor neurons or muscle potentials preferably simultaneously with the responses of the corresponding upstream LPTCs the contribution of postsensory sources of variability could in future be unravelled in more detail.

Despite a considerable amount of variability that we conclude adds to the signals inherited from the VS2/3-cells, head pitch responses seem to be executed more reliably than steering responses, the time course of which could only be reproduced in model simulations by incorporating 16 times the noise of an LPTC [15]. A higher reliability of head movements than of steering responses is what one would expect taking into account the fact that head movements of flies fine-tune gaze stabilisation in defined periods of locomotor activity [53], [54]. The reliability of visually mediated head movements may even be higher under real world conditions, when they are mediated by combining the information from several sensory modalities (see above and [55], [56]).

The impact of noise sources arising within an information processing pathway from the sensory periphery to the final behavioral outcome has previously been addressed (see above). Recently, variability was also found to increase at consecutive levels of processing in the auditory system of grasshoppers [57], [58]. Additionally, similar to our findings, postsensory noise of about the same amplitude as sensory noise can explain the variability of human eye movements [3], [4] (see however [2]). Hence, despite neuronal mechanisms that may have evolved to optimize information transmission, variability accumulates in several species along a given sensory-motor pathway.

## Materials and Methods

### Electrophysiology

One to four days old female blowflies (*Calliphora vicina*) were taken from the laboratory stock. The animals were mounted on custom-made holders. Wings and legs were removed and wounds sealed by bees wax. In five of seven flies one haltere was fixated, allowing only the other haltere to oscillate (see below). In the remaining two flies both halteres were free to move. Movements of a haltere were used as indicators for the activity state [16], [17]. They oscillate when flies are walking or flying [30]. A small hole was cut into the head capsule to allow access to the lobula plate. We recorded intracellularly from seven VS2/3-cells in seven different flies. These cells are sensitive in the frontal part of the visual field of the fly and depolarise in response to downward motion [24], [59]. VS2/3-cells are thought to mediate downwards directed head pitch movements [25], [26]. For the recording we used borosilicate micropipettes drawn on a microelectrode puller (Sutter Instruments P-97). Electrodes were filled with 1 M KCl and had a typical tip resistance of 25 to 60 MOhm. Data were recorded using a 16 bit A/D-converter (DAQBoard 2000, IOtech, Cleveland, OH) and analysed off-line with MATLAB (The MathWorks Inc., Natick, MA, USA).

### Image data acquisition and evaluation

During neuronal recordings of five VS-cells (in five different flies) we monitored the activity of one haltere to distinguish the state of motor activity of the flies using a CMOS camera (LOGLUX i5CL, Kamera Werk Dresden, Dresden, Germany) running at 250 Hz. The images were acquired using a National Instruments frame grabber (National Instruments, Austin, TX, USA) and a standard PC. The flies were illuminated by near-infrared light-emitting diodes with a peak wavelength of 870 nm which is beyond the spectral sensitivity of *Calliphora* photoreceptors [60], [61]. The spectral sensitivity of the camera ranged up to 1000 nm. We painted the haltere tips by infrared reflecting dye to facilitate analysis of haltere movements. The two halteres can beat independently [16], and often one haltere starts oscillating several milliseconds before the other haltere does (Rosner, unpublished results). Since they do not always beat at the same time it is favourable to record oscillations of both halteres to gain information about the fly's state of motor activity. However, in our experimental setup space was very restricted allowing us to film one haltere only. We fixated the haltere which was not filmed. Thus, we cannot be sure that all data we acquired without observable haltere oscillation were indeed acquired during a state of reduced motor activity. However, we can say for sure that trials with haltere oscillation were recorded during states of elevated motor activity. Haltere beat was evaluated offline by visual inspection and, additionally, as described in detail in [16].

The data on head pitch movements of the flies were acquired in a previous study [16]. Data acquisition and evaluation are described there in detail. However, we simplified the analysis in two regards by readjusting evaluation time windows: (i) As described in [16] only a subset of trials was selected for further analysis to minimize a potential effect of variable head position at the start of pattern motion on the final pitch response. We accepted only those trials for further analysis with a head orientation that deviates by less than 5 deg. from a reference orientation (for definition of reference orientation see [16]). For the present study we readjusted the time interval for determining these head orientations by calculating the mean across a 30 ms interval starting 20 ms before and ending 10 ms after stimulus motion onset. (ii) The measured head orientation traces of all selected trials were aligned to have zero mean in the same time window starting 20 ms before and ending 10 ms after the onset of stimulus motion. This procedure was applied to each fly separately. Both readjustments served to standardize the time windows, but do not affect the conclusions drawn in the previous account.

In the behavioral experiments the halteres served only indirectly as the indicator of the activity state. Instead head jitter movements were found to be highly correlated with haltere movements and served as the indicator of the behavioral state [16].

### Visual stimulation

The visual motion stimulus applied in the electrophysiological experiments of the present account was identical to the stimulus which was presented to flies in behavioral experiments in a previous study [16]. Flies were mounted in front of a CRT monitor with a resolution of 640×480 pixels and a refresh rate of 240 Hz. The stimuli were programmed and presented utilising a Visage stimulus generator (Cambridge Research Systems, Cambridge, UK), Matlab and a standard PC. The monitor was positioned symmetrically in front of the fly enabling the stimulus pattern to span an elevation from –25 deg. (ventral) to +45 deg. (dorsal) and an azimuth from −45 deg to +45 deg. with respect to a straight head position of the fly (0 deg., 0 deg.). We used a random dot pattern as the visual stimulus. The pattern consisted of 40 randomly positioned bright dots (65 cd/m^2^) of 2 deg. horizontal and 2 deg. vertical extent in front of a dark background (0.0 cd/m^2^). In each experiment, the same stimulus was presented repeatedly to a fly. During each trial in an electrophysiological experiment, the stimulus pattern moved downwards for 200 ms at 168 deg. s^−1^ after a stationary pattern had been shown for 1000 ms. The stationary pattern was preceded by a 300 ms flicker stimulus. The flicker did not serve any particular purpose in the electrophysiological experiments, but was applied in the behavioral experiments to reset the head position after the preceding trial. To employ the same stimulus conditions in the electrophysiological and the behavioral experiments we presented the reset stimulus in both cases. In the behavioral experiments not all flies experienced flicker as reset stimulus, some experienced upwards directed motion. We do not have any indication that any specific reset stimulus affects the signal-to-noise ratio (SNR) of the behavioral experiments.

### Modelling

To compare the variability of head optomotor movements with the variability of neuronal responses we applied a simple modelling approach. We predicted head movements from neuronal recordings and compared the variability of predicted and actually observed head movements. For predicting head movements we applied either a first-order low-pass filter or a second-order low-pass filter to the neuronal recordings, depending on whether we aimed to simulate head movements of a fly being in a state of elevated or reduced motor activity (see Results section). The filter windows were 2600 ms in length. The first-order low-pass filters had time constants of 50 ms, 100 or 150 ms. The time constants of the second-order low-pass filters comprised all combinations of the three time constants used for the first-order filters.

### Reliability analysis

To compare the reliability of actually observed head pitch movements with the reliability of the neuronal responses we determined the signal-to-noise ratio (SNR) of the observed head movements and the predictions based on the neuronal responses. For this we calculated the mean across trials for each single fly and divided the mean by the corresponding standard deviation. To reduce variability originating from variable recording quality, we only used sequences of trials with a rather stable resting potential not deviating by more than 2.5 mV from the other trials of the same sequence. The resting potential was determined from the average across 1.5 s prior to visual stimulation when the monitor was dark. Moreover a sequence of at least seven successively recorded trials was required for further analysis. For some flies we evaluated more than a single sequence of consecutive trials. In these cases we calculated the SNR for each individual sequence of consecutive trials and then determined the mean SNR for that particular fly across the SNRs determined for the individual sequences.
